# Time-resolved diffraction of shock-released SiO_2_ and diaplectic glass formation

**DOI:** 10.1038/s41467-017-01791-y

**Published:** 2017-11-14

**Authors:** A. E. Gleason, C. A. Bolme, H. J. Lee, B. Nagler, E. Galtier, R. G. Kraus, R. Sandberg, W. Yang, F. Langenhorst, W. L. Mao

**Affiliations:** 1 Shock and Detonation Physics, Los Alamos National Laboratory, P.O. Box 1663, Los Alamos, NM 87545 USA; 2 Stanford Institute for Materials and Energy Sciences, SLAC National Accelerator Laboratory, 2575 Sand Hill Rd., Menlo Park, CA 94025 USA; 3 Linac Coherent Light Source, SLAC National Accelerator Laboratory, 2575 Sand Hill Rd., Menlo Park, CA 94025 USA; 4 Shock Physics, Lawrence Livermore National Laboratory, 7000 East Ave., Livermore, CA 94550 USA; 5 Center for Integrated Nanotechnologies, Los Alamos National Laboratory, P.O. Box 1663, Los Alamos, NM 87545 USA; 6 Center for High Pressure Science and Technology Advanced Research, Shanghai, 201203 China; 7 HPSynC, Carnegie Institution of Washington, Argonne, IL 60439 USA; 8 Institut für Geowissenschaften, Friedrich-Schiller-Universität Jena, D-07745 Jena, Germany; 9 Geological Sciences, Stanford University, 367 Panama St., Stanford, CA 94305 USA

## Abstract

Understanding how rock-forming minerals transform under shock loading is critical for modeling collisions between planetary bodies, interpreting the significance of shock features in minerals and for using them as diagnostic indicators of impact conditions, such as shock pressure. To date, our understanding of the formation processes experienced by shocked materials is based exclusively on ex situ analyses of recovered samples. Formation mechanisms and origins of commonly observed mesoscale material features, such as diaplectic (i.e., shocked) glass, remain therefore controversial and unresolvable. Here we show in situ pump-probe X-ray diffraction measurements on fused silica crystallizing to stishovite on shock compression and then converting to an amorphous phase on shock release in only 2.4 ns from 33.6 GPa. Recovered glass fragments suggest permanent densification. These observations of real-time diaplectic glass formation attest that it is a back-transformation product of stishovite with implications for revising traditional shock metamorphism stages.

## Abstract (web summary)

Our understanding of shock metamorphism and thus the collision of planetary bodies is limited by a dependence on ex situ analyses. Here, the authors perform in situ analysis on shocked-produced densified glass and show that estimates of impactor size based on traditional techniques are likely inflated.

## Introduction

During a collision between two bodies, e.g., the impact of an asteroid with the Earth, rocks are suddenly subjected to very high pressures and temperatures resulting in so-called shock metamorphism^1,2^. Our understanding of shock metamorphism is still quite incomplete, derived only from ex situ investigation of experimental samples, studies of naturally impacted materials, and theoretical analyses (e.g., refs. ^3–5^). Properties of shock-metamorphosed material are commonly derived from time-infinity (i.e., recovered) natural samples probed using petrographic microscopy, vibrational spectroscopy, transmission electron microscopy, and synchrotron-based diffraction analyses (e.g., refs. ^6–11^). A naturally sourced shock-recovered sample has undergone thermodynamically different compression loading and unloading paths during the passage of the initial shock and subsequent release waves. Yet, the phase transition mechanisms, timing, and conditions of formation for specific features in shock-effected minerals, e.g., amorphous lamellae, diaplectic glass (i.e., shocked-produced densified glass) and high-pressure phases, routinely used as impact event barometers are largely unknown. Only recently^8,12^ have scientists been able to provide estimates on the shock-release timescale and peak pressure-temperature conditions for formation of shock melt vein material. In particular, there are open questions regarding the transformation pathway and pressure-temperature range of formation for diaplectic silica (SiO_2_) glass as a bulk glass or in amorphous lamellae as planar deformation features—the best-studied mineralogical shock barometer. SiO_2_ has been extensively studied in both the static and dynamic compression communities (e.g., refs. ^13–15^). While static compression experiments on quartz show evidence of on-compression pressure-induced amorphization above ~30 GPa, e.g., ref. ^16^, the so-called ‘mixed phase region’ (>20 GPa) of the principle Hugoniot (i.e., locus of all possible thermodynamic states behind a shock wave) is traditionally interpreted to reflect the transformation to stishovite, which reverts then back to glass^4^. However, this interpretation is questioned due to the reconstructive nature of the quartz-to-stishovite transition and the expected sluggish kinetics^17^. Therefore knowledge of the pressure-temperature-time path and time-resolved in situ diffraction measurements of experimentally shocked samples are required to understand the formation of diaplectic glass and its significance for naturally shocked samples.

Here we examine the shock release behavior of SiO_2_ after formation of stishovite using the Linac Coherent Light Source (LCLS) X-ray Free Electron Laser (XFEL) combined with laser-driven shock compression. We report results of amorphization of SiO_2_ on shock-release from stishovite. Debye–Scherrer patterns were recorded during the release of the shock wave through an initially fused silica (SiO_2_) sample. Temporally resolved X-ray diffraction (XRD) patterns clearly demonstrate the metastability kinetics of stishovite converting into an amorphous state below the melt temperature, provide constraints on the formation mechanism of diaplectic glass, revise down the pressure limit of formation to 30 GPa and add a temporal dimension to the progressive stages of shock metamorphism.

## Results

### Diffraction on release

**Fig. 1:**
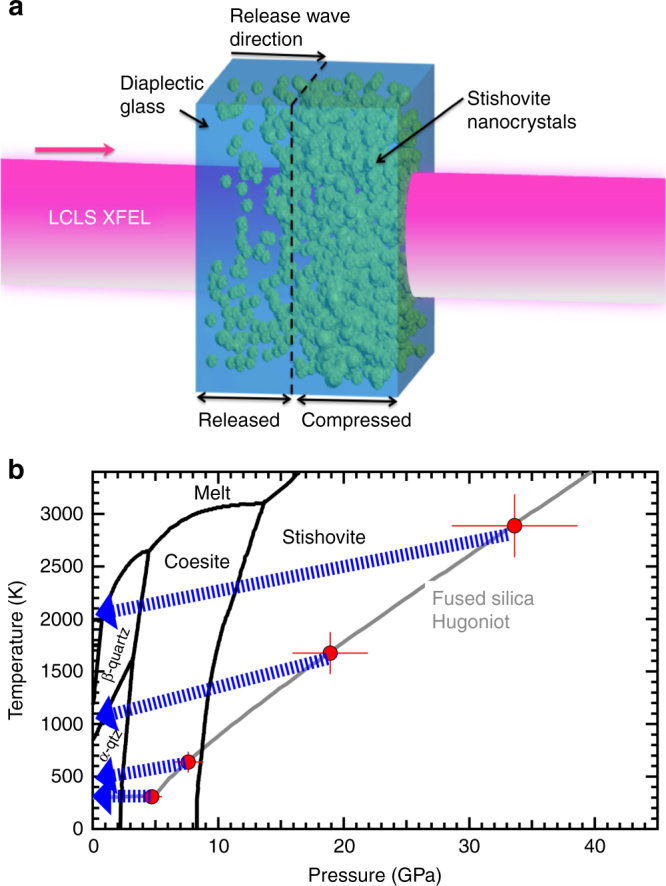
Experimental configuration and explored phase space. **a** Target schematic for sample during the shock-release process. During the onset of release, newly formed grains of stishovite (green sphere-like features) dissolve over a few nanoseconds leaving behind diaplectic glass. **b** Equilibrium phase diagram^32^ of SiO_2_ showing high-pressure polymorphic phase boundaries and melt curve (black). The fused silica Hugoniot (gray) using data^18,33,34^. Red points are maximum pressure, temperature conditions achieved for particular ablation drive laser parameters as determined from velocimetry records. The error bars include scatter in the measured transit times, uncertainty in the total sample thickness, uncertainty in the pressure-irradiance scaling law^18^. Isentropic release paths (blue arrows, determined using Sekine et al.^35^) show the approximate conditions achieved in this experiment at late time delays, e.g., 12–30 ns (i.e., during release), and release shock temperatures are determined from post shock temperatures for fused silica^36^

Using an experimental setup similar to Gleason et al.^18^, we explore the changes in the atomic structure of SiO_2_ using transmission in situ XRD with 8 keV X-rays from the XFEL at the Matter in Extreme Conditions (MEC) end-station of the LCLS. Laser ablation from a frequency-doubled Nd:Glass laser system was used to launch a compressive (or shock) wave over the pulse duration, 10 ns. It takes ~10–11 ns for this compressive wave to traverse the sample. Therefore any XRD pattern collected at an X-ray probe time longer than this will include a sampling of material experiencing release (Fig. 1a) due to completion of shock transit and drive laser cessation. Diffraction data presented here were collected during the passage of a shock-release wave. Phase space accessed in this experiment (Fig. 1b) shows the quasi-isentropic release paths and approximate pressure–temperature conditions achieved between 12 and 30 ns.

**Fig. 2:**
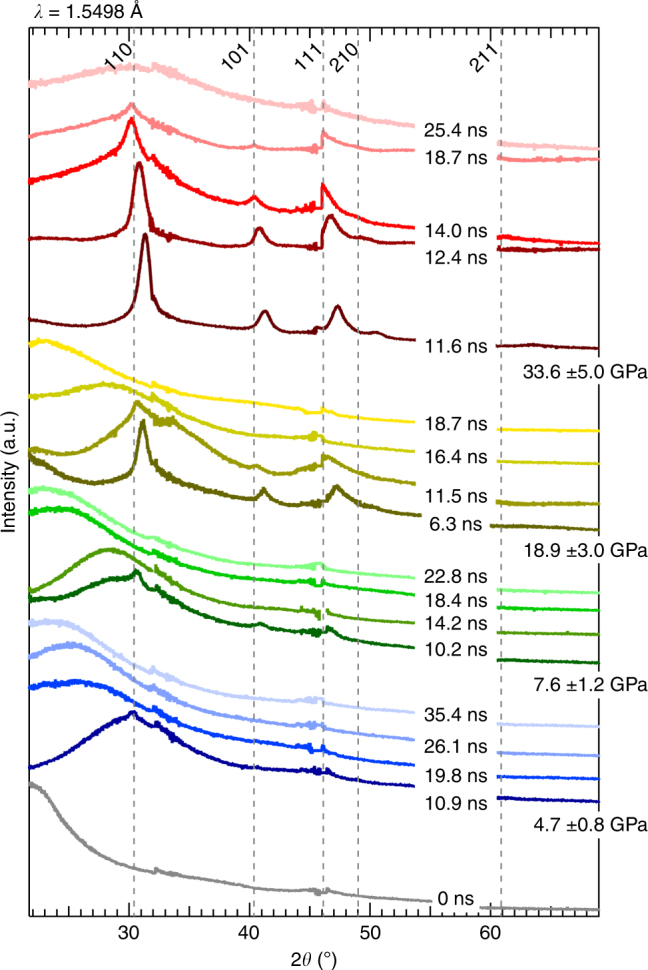
Multiplot of XRD data. Stishovite peaks are labeled at the top; ambient condition positions (gray dashed lines). Traces are clustered according to maximum applied pressure with time delays listed on shock-release. Offset along the *y* axis and color scheme of the traces are arbitrary to enable viewing clarity. Discontinuities in the traces are seen at 32.5°, 46.0° and 58.0° 2*θ* due to spacing between the mosaicked active areas of the detectors

The first sharp diffraction peak (FSDP) from ambient fused silica (starting density, 2.20 grams per cubic centimeter) is centered at 2*θ* ~21.6^o^ (Fig. 2, gray curve), consistent with previous work (e.g., ref. ^19^). For each peak pressure set, 33.6 ± 5.0, 18.9 ± 3.0, 7.6 ± 1.2, and 4.7 ± 0.8 GPa, XRD patterns are collected on release (i.e., at time delays greater than ~11 ns), Fig. 2. XRD traces collected on release show phases inherently transitioning to lower pressure. Previous work^18^ shows fused silica transforming to randomly oriented, nanometer-sized grains of stishovite on compression. However, in the lowest two pressures, 4.7 and 7.6 GPa, diffraction shows super-positioning of stishovite peaks with a strong diffuse signal. This diffuse feature is interpreted to reflect the random network of compressed amorphous SiO_2_. At these pressures on release, the stishovite grains are no longer resolvable, and the diffuse feature progressively shifts to lower 2*θ* indicating the glass is at a lower pressure state at each sequential time-slice. At 18.9 GPa, the 6.3 ns trace shows fused silica converts to stishovite on compression and a diffuse signature centered near 22° 2*θ* confirming the velocimetry data that the compressive wave has not yet transited the entire sample. For this pressure, we do not have data at the moment of final compression (i.e., ~10 ns). On release from 18.9 GPa stishovite peaks first shift to lower 2*θ* as the signal of the diffuse feature increases. We interpret this diffuse feature again as a compressed glass, which then progressively shifts to lower 2*θ* with decreasing pressure. Using a method of background subtraction and normalization^19^ to estimate phase fraction amorphous vs. crystalline, we find the final compression, highest pressure trace shows full conversion to stishovite on compression and the crystallinity persists for at least 7 ns after onset of release. This 7 ns is markedly longer than any other pressure set which show stishovite crystals only persisting for a few to fractions of a nanosecond. At ~4 ns after onset of release there is an increase in signal of a diffuse feature reflecting the random network structure of a compressed glass. The peak position of this diffuse feature does not change as pressure decreases.

XRD collected at time infinity (i.e., from recovered debris) still shows a diffuse signal with an FSDP position recording a smaller *d*-spacing than the starting fused silica, indicative of a compressed amorphous material (Supplementary Fig. 1). The fused silica Hugoniot is plotted in pressure-entropy space (Supplementary Fig. 2) to determine what peak shock pressures intersect the liquidus. We find our highest pressure point of 33.6 GPa may cross the melt bound on release, therefore we cannot rule out a melt state as a contributor to the diffuse signal seen at 25.4 ns; see Supplementary Discussion.

**Fig. 3:**
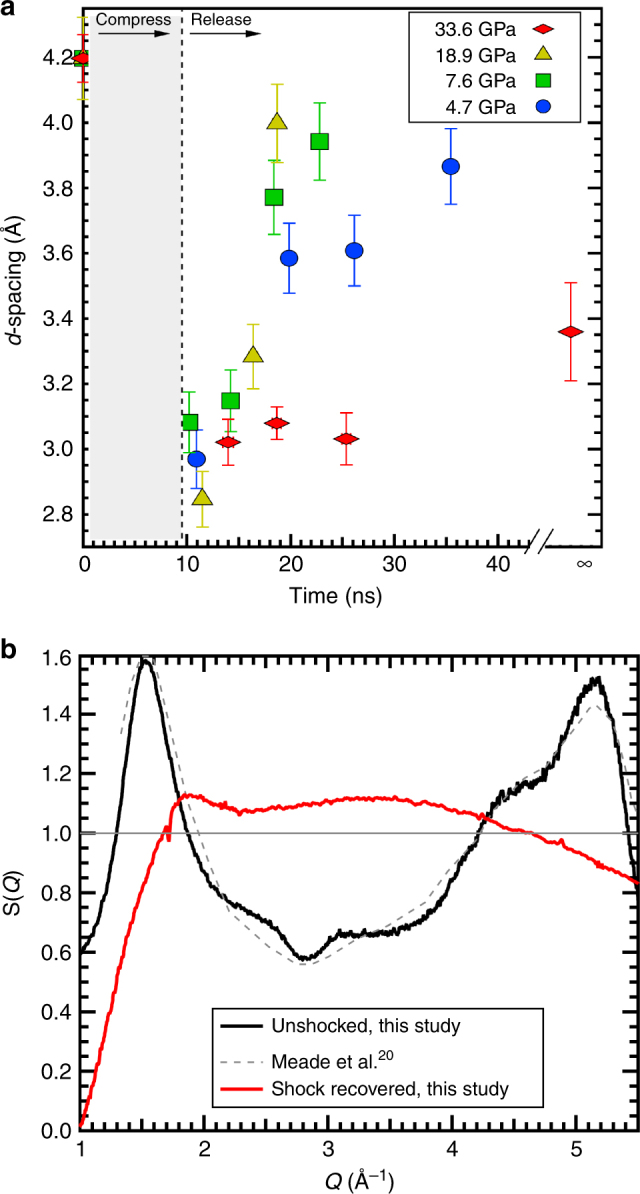
Time-dependence and S(*Q*) for first sharp diffraction peak. **a** For each trace, corresponding to a time delay within a given pressure data set, the peak center of the first sharp diffraction peak *d*-spacing is measured from the raw data, where the error bar indicates one standard deviation for the Gaussian profile fit. The trend for the lowest three pressures appears to return to a *d*-spacing similar to the original fused silica starting material. However, the highest pressure, time-infinity trace (Supplementary Fig. 2) from the recovered material shows a smaller *d*-spacing consistent with a trend consistent with a (possibly) higher density diaplectic glass. **b** The X-ray structure factor, S(*Q*) for the starting material fused silica (black curve) and recovered material (red curve), both collected at 25 keV, is used to determine the real space correlations in the glass

If the peak positions from the amorphous material FSDPs collected on release are plotted as a function of time, there is a striking trend difference between the lowest three pressures, up to 18.9 GPa, compared to the highest pressure, 33.6 GPa (Fig. 3a). Between 4.7–18.9 GPa immediately at onset of release, the FSDP *d*-spacing decreases sharply at a rate of ~0.05 Å/ns, trending toward the starting FSDP position of ambient fused silica 4.20(1) Å. However, the 33.6 GPa data show a nearly constant FSDP at 3.1 Å up to 25 ns after the onset of release. The FSDP of amorphous shock recovered debris from 33.6 GPa is at a smaller *d*-spacing, 3.36(2) Å, compared to that of the starting material. The X-ray structure factor S(*Q*) for the starting fused silica and the recovered material was determined from XRD collected at 25 keV (Beamline 12.2.2, ALS) (Fig. 3b). Ambient condition S(*Q*) compares well with previous work^20^. The average pair correlation functions, G(r) for these data (Supplementary Fig. 3) are obtained from the Fourier sine transform of S(*Q*). Though G(r) does not provide a direct measure of Si-O coordination, it can constrain the nearest-neighbor bond lengths. Ambient starting fused silica shows a <Si-O> bond distance of 1.58(2) Å consistent with a 4-fold coordinated glass. Interestingly, the shock recovered material shows a <Si-O> bond distance of 1.68(5) Å—a marked increase in length consistent with a mixture of 4 and 6 coordination^5,21,22^.

## Discussion

**Fig. 4:**
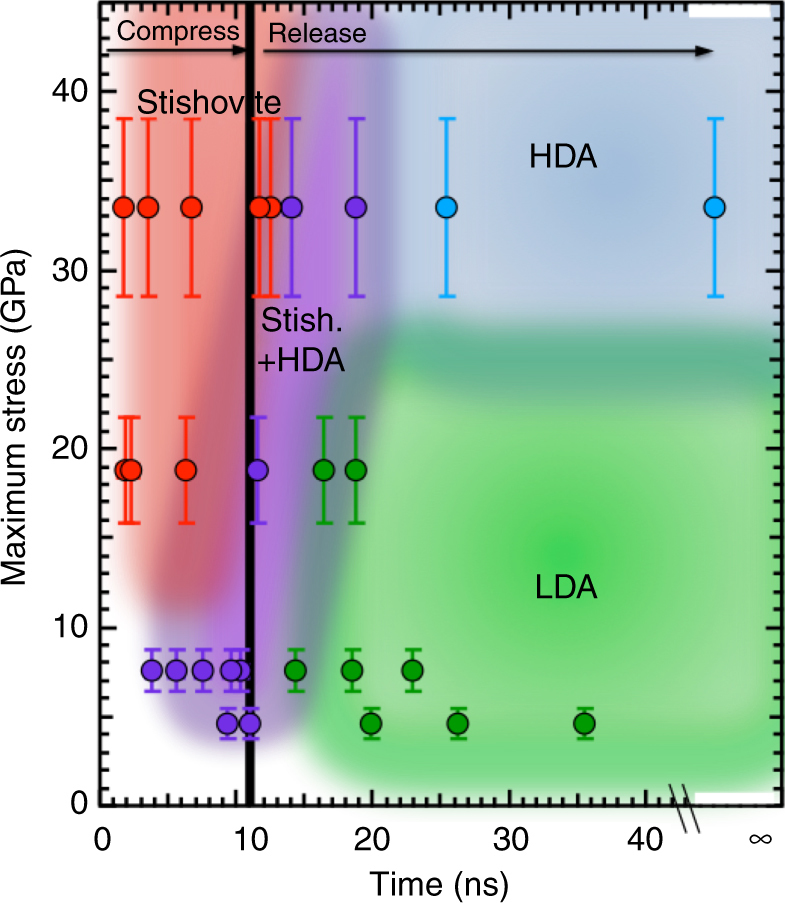
Reaction diagram of SiO_2_ under shock compression. Metastabilty phase diagram showing peak pressure phase as a function of time from compression and release path. Colored area approximately cluster similar phases to show pressure (and associated shock temperature)—time boundaries. Stress error bars are determined from velocimetry and include scatter in the measured transit times, uncertainty in the total sample thickness, uncertainty in the pressure-irradiance scaling law^18^

The formation process of this resultant amorphous state does not require crossing the melt boundary^23^ and can either have a FSDP position that trends back to the starting (larger) low-density amorphous (LDA) fused silica position, or retains a smaller relative FSPD *d*-spacing, classified as high-density amorphous (HDA), Supplementary Discussion. This definition is supported by observations of naturally formed diaplectic glasses, which show a distribution of densities (e.g., refs. ^24, 25^). From the 4.7 and 7.6 GPa release diffraction data we cannot resolve any structural change in the amorphous material, yet we do see the starting amorphous material compress, transform a small volume to stishovite, between 0.5 and 7%, and then release to an amorphous state. Due to data resolution (i.e., limited *Q*-space coverage), assignment of a different amorphous structure after release for these data would be an over-reach. Therefore, we cannot classify material formed on release from 4.7 and 7.6 GPa as diaplectic glass, corroborating the findings from static compression experiments, e.g., refs. ^20,26,^. However, data collected on release from 18.9 and 33.6 GPa do show amorphization after conversion to stishovite, therefore we can assign the final state to be a diaplectic glass. We map out the pressure-temperature-time-phase space of SiO_2_ from Gleason et al.^18^ and this study using the above classifications (Fig. 4). As a function of peak pressure (and therefore temperature) and time, we identify trends in explored transient states (color coded in Fig. 4). Interestingly, stishovite+ HDA is seen to persist over a very narrow pressure-time space, and there may be a threshold pressure of ~25 GPa to lock in an HDA-like diaplectic glass.

Since our loading (and unloading) path is designed such that the sample experiences the passage of a single compressive wave, our in situ data are more representative of a natural shock event than the traditional reverberation shock scheme (e.g., ref. ^27^) employed by gas gun or high-explosive sample recovery experiments. Moreover, we are now able to add temporal dimension to the progressive stages of shock metamorphism^28,29^, moving from a peak pressure phase classification to a pressure-time-phase classification, which inherently enables identification of formation mechanism. Currently, using the stages of progressive shock metamorphism that were established by occurrence of diagnostic shock features in recovered samples, the onset of diaplectic glass formation is thought to be at Stage II, from peak pressure ~35–45 GPa. On the basis of our data, we find it is the entropy state that defines the peak pressure required to form diaplectic glass on release^30^. Fused silica shocked to 19 GPa on the Hugoniot reaches the same entropy state as a 30 GPa shock in quartz. Therefore, in quartz or a quartz-rich material, 30 GPa is the peak pressure needed to form diaplectic glass on release—revising down the Stage II pressure threshold by ~25%. The relationship between peak pressure, as determined from shock-metamorphosed mineral observations, and impactor size is inflated by 25%. This requires revision of the Shock Stages if the pressures needed for diaplectic glass recovery are actually lower than originally thought and provides a new and important constraint on planetary formation processes and modeling. Additional insights as to why stishovite reverts to an amorphous state after conversion in its phase stability field on shock release may be due to limited thermal stability as demonstrated by recent work^31^.

## Methods

### Experimental Setup

XRD from each pump-probe experiment, recorded on the Cornell-SLAC Pixel Array Detectors (CSPADs) was azimuthally integrated as a function of X-ray scattering angle (2*θ*), see Gleason et al.^18^: Methods section. The applied pressure, *P*, from laser ablation was determined using the known fused silica principal Hugoniot^37^ and shock speed (Gleason et al.^18^: Supplementary Discussion, VISAR Analysis Details). Applied pressures of 33.6 ± 5.0, 18.9 ± 3.0, 7.6 ± 1.2, and 4.7 ± 0.8 GPa were set by the incident laser intensity. XRD measurements were spatially integrated over the whole sample and therefore the diffraction measures varying contributions from peak pressure state and evolving phase on release. Time zero was defined as the time when the shock wave enters the fused SiO_2_.

We used the Matter in Extreme Conditions (MEC) instrument at the Linac Coherent Light Source (LCLS)^38^, and quasi-monochromatic (d*E*/*E* = 0.2–0.5%), 7.952(30) keV X-ray pulses of 60 fs duration with an average of ~10^12^ photons per pulse to probe our target package. LCLS X-ray free electron laser spot size was 75 um diameter. Wafers of amorphous Nikon synthetic fused silica (SiO_2_) were double-side parallel polished to a thickness of 60 μm and diced into 2 × 2 mm individual targets. These targets were batch-coated with 10 μm of plastic (glow discharge polymer deposition of trans-2-butene, 1 C:1.3 H, (ref. ^39^)) to serve as the ablator. Using phase plates on the optical drive laser, a 200 μm diameter flat-top laser spot was used to achieve focal spot intensity of ~ 10^12^ W/cm^2^. An ablation-driven compression wave was launched parallel to the sample normal using a 10 ns quasi-square pulse profile from a frequency-doubled Nd:Glass laser system (*λ* = 527 nm). The optical laser and X-ray beam were spatially overlapped and operated in single shot mode. The absolute time zero corresponds to overlap of their leading edges. For each shot, a time delay was selected for the XFEL pulse relative to the optical laser pulse with a jitter of 0.3–0.5 ns; included in Figs 3 and 4. We establish a relative time zero defined as the time at which the pressure wave reaches the interface between the plastic ablator and the SiO_2_. The transit time through the plastic ablator varies as a function of drive energy and was determined from VISAR measurements^18^. The combined use of a pressure-irradiance scaling and the transit time provided constraints on the applied pressure for each shot. The pump-probe delay scans at several nanosecond intervals enabled collection of a time-series of XRD patterns in transmission geometry. XRD patterns were captured by Cornell-SLAC Pixel Array Detectors (CSPADs) constructed of individual application-specific integrated circuits (ASICs)^40^. Maximum azimuthal angle coverage was 23°. One target was shot per selected time delay.

Recovered material from a single shot was captured in Lexan plastic placed downstream of the sample. Shot debris was removed from the Lexan and placed in a sandwich of single crystal diamond platelets. These platelets were mounted on a metal gasket to allow for sample-to-detector distance determination at the 12.2.2 Advanced Light Source (ALS) Beamline^41^ to collect XRD of the debris at *λ* = 0.4959 Å. Separate measurements of Lexan alone, starting fused silica sample material, and diamond platelets+air were recorded to reference background and possible sources of contamination (e.g., signal from Lexan or unshocked fused silica).

### Data availability

All relevant data are available from the authors upon request.

## Electronic supplementary material

Supplementary Information
