# Supplementary material for: Time-resolved diffraction of shock-released SiO2 and diaplectic glass formation
Source: Nat Commun. 2017 Nov 14;8:1481. doi: 10.1038/s41467-017-01791-y (PMC5684137; doi:10.1038/s41467-017-01791-y)
Supplement: Supplementary file 1 — Supplementary Information [file 41467_2017_1791_MOESM1_ESM.pdf]

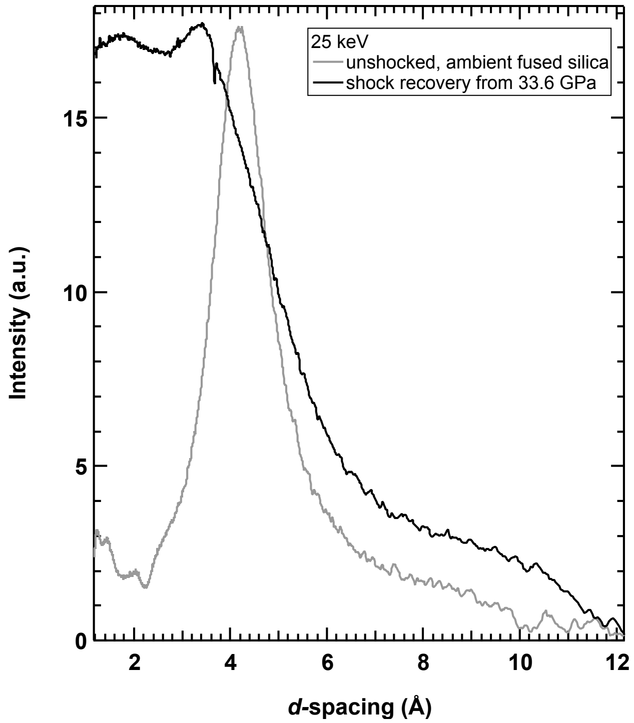

Supplementary Figure 1. **X-ray diffraction from recovered fused silica sample shock compressed up to 33.6 GPa.** A 5 mm thick Lexan substrate was placed immediately downstream of the target (no glue layer) to 'catch' debris. Recovered material was extracted from the Lexan substrate and X-rayed at the Advanced Light Source, LBNL, beamline 12.2.2. Using 25 keV, we find the first sharp diffraction peak (FSDP) of our recovered material to be at 3.36(2) Å. As a crosscheck, the FSDP of Lexan alone is at 6.88 Å.

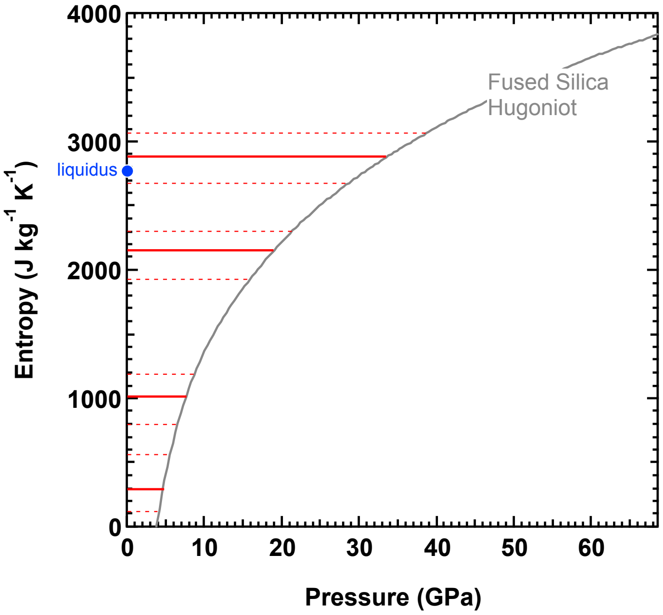

Supplementary Figure 2. **Fused silica Hugoniot in entropy-pressure space**[1,2]**.** Solid red lines indicate approximate release paths taken from peak pressures 33.6, 18.9, 7.6 and 4.7 GPa. Dashed red lines are the uncertainty envelopes derived from uncertainty in the peak pressure as determined in Gleason et al.[3]. See Supp. Mat. Discussion 1 on fused silica pressure-entropy space.

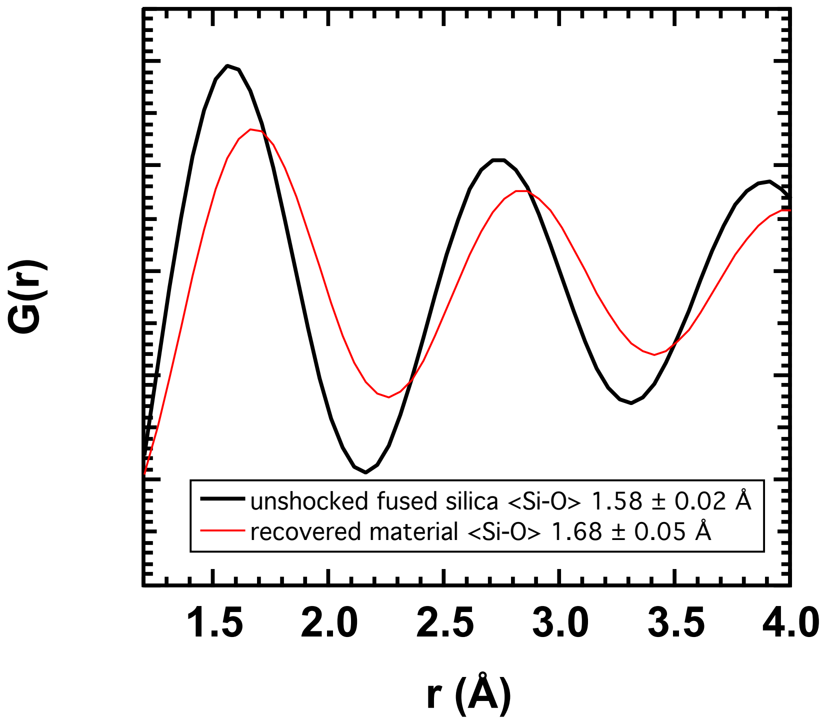

Supplementary Figure 3. **Average pair correlation functions G(r).** Using the Fourier sine transform listed in Meade et al.[4]: $G(r) = \frac{2}{\pi}\int Q[S(Q) - 1]\sin(Qr)\,dQ$, where $Q = 4\pi\sin\theta/\lambda$, we determine the real space correlations in the ambient starting fused silica glass and in the shock recovered glass. The first dominant feature is taken to indicate the <Si-O> bond distance. To calculate the S(Q), data corrections include parasitic scatter from the sample holder and air scatter. Each were collected as separate patterns at BL 12.2.2, ALS and subtracted (point-by-point) after integration over the same 2theta and azimuthal range from the sample to correct for any elastic diffuse scattering. Compton scattering was negligible.

Supplementary Discussion

Fused silica pressure-entropy space. The melting temperature of Nikon synthetic fused silica is 1858 K (Nikon Corp. personal comm.). Richet et al.[5] gives the entropy at this temperature, ambient pressure S^o_T=166.54 J/mol/K (interpolated between 1800 and 1900K on Richet et al.[5]'s Table 12 for 'Thermodynamic properties of amorphous SiO2'. Using a molar mass of 0.06009 kg/mol we convert to S^o_T= 2771.53 J/kg/K (blue dot, liquidus). This is order of magnitude similar to values calculated in Refs. 6,7, albeit at lower temperatures. The uncertainty envelope for our highest pressure release isentrope intersects the liquidus point. This brings into question the nature of the diffuse feature seen in the diffraction for this time series. In XRD a liquid melt and amorphous solid would have the same diffuse character, though likely peak positions at different *d*-spacings and perhaps different peak widths. However, from our XRD data we cannot resolve this difference.

Amorphous material categories. If the FSDP from amorphous material shows a 50% reduction in *d*-spacing, we categorize that as a high-density amorphous (HDA) state, even if transient (i.e., recorded via XRD as persisting for only a few nanoseconds). A *d*-spacing reduction of less than 50% is categorized as a low-density amorphous (LDA) state, assumed to have a similar structure to the starting fused silica. Here, we define diaplectic glass as a glass formed after a sample has fully transformed to stishovite, i.e., undergone a reconstructive phase transformation, and then returned to an amorphous state.

Supplementary Materials References

1. Boslough, M. Post-shock temperatures in silica, *J. Geophys. Res.* **93**, 6477-6484, 1988.

2. Akins, J. & Ahrens, T. Dynamic compression of SiO2: a new interpretation. *Geophys. Res. Lett.* **29**, 1394, 2002.

3. Gleason, A. *et al.* Ultrafast visualization of crystallization and grain growth in shock-compressed SiO2. *Nat. Comm.* **6**, 8191 (2015).

4. Meade, C., Hemley, R. & Mao, H.K. High-pressure x-ray diffraction of SiO_2 glass. *Phys. Rev. Lett.* **69**, 1387-1390 (1992).

5. Richet, P., Bottinga, Y., Denielou, L., Petitiet, J., & Tequi, C. Thermodynamic properties of quartz, cristobalite and amorphous SiO_2: drop calorimetry measurements between 1000 and 1800 K and a review from 0 to 2000 K. *Geochimica et Cosmochimica Acta* **46**, 2639-2658 (1982).

6. Melosh, H.J. A hydrocode equation of state for SiO2. *Meteor. Planet. Sci.* **42**, 2079-2098 (2007).

7. Kraus, R. *et al*. Shock vaporization of silica and the thermodynamics of planetary impact events. *J. Geophys Res*. **117**, E09009 (2012).
